# Tumor-directed immunotherapy can generate tumor-specific T cell responses through localized co-stimulation

**DOI:** 10.1007/s00262-016-1909-3

**Published:** 2016-10-06

**Authors:** Peter Ellmark, Sara M. Mangsbo, Christina Furebring, Per Norlén, Thomas H. Tötterman

**Affiliations:** 1 Alligator Bioscience AB, Medicon Village, 223 63 Lund, Sweden; 2 Department of Immunotechnology, Lund University, Lund, Sweden; 3 Department of Immunology, Genetics and Pathology, Uppsala University, Uppsala, Sweden

**Keywords:** Immunotherapy, Tumor-directed immunotherapy, Cancer, Intratumoral, Bispecific antibody, Immuno-oncology

## Abstract

The most important goals for the field of immuno-oncology are to improve the response rate and increase the number of tumor indications that respond to immunotherapy, without increasing adverse side effects. One approach to achieve these goals is to use tumor-directed immunotherapy, i.e., to focus the immune activation to the most relevant part of the immune system. This may improve anti-tumor efficacy as well as reduce immune-related adverse events. Tumor-directed immune activation can be achieved by local injections of immune modulators in the tumor area or by directing the immune modulator to the tumor using bispecific antibodies. In this review, we focus on therapies targeting checkpoint inhibitors and co-stimulatory receptors that can generate tumor-specific T cell responses through localized immune activation.

## Introduction

The groundbreaking results with CTLA-4 and PD-1/PD-L1 checkpoint blocking antibodies provide a solid foundation for the field of cancer immunotherapy to build on. The field is now geared toward identifying drug candidates that act complementary or synergistically with checkpoint inhibitors to enhance the response rates [1]. At the same time, treatments need to be safer in order to allow a broader use of cancer immunotherapy.

**Fig. 1:**
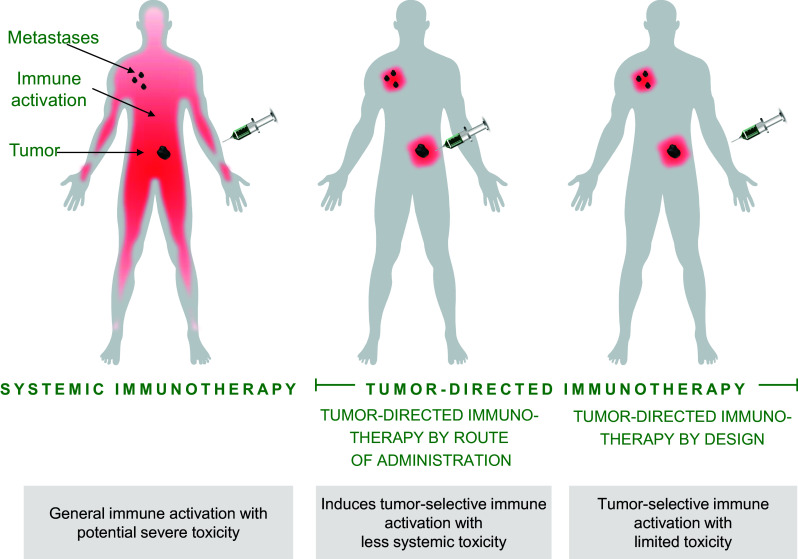
Illustration of tumor-directed immunotherapy (also termed in situ vaccination) compared to systemic immunotherapy. Intravenous administration of agonistic or checkpoint blocking antibodies activates tumor-directed T cells generating an anti-tumor response. However, these treatments can also induce cytokine release, cause liver problems, and activate autoreactive T cells, resulting in immune-related adverse events. Tumor-directed immunotherapy aims to direct immune activation to the tumor and tumor-draining lymph node axis. Activated tumor-directed T cells have the potential to migrate to distant tumors, eradicating also metastatic lesions. In contrast to systemic immunotherapy, the impact on immune cells irrelevant for the anti-tumor response is reduced. There are two approaches to tumor-directed immunotherapy: tumor-directed immunotherapy by administration route and tumor-directed immunotherapy by design. Tumor-directed immunotherapy by administration route is achieved by administering the immunomodulatory antibody directly into the tumor, into tumor-draining lymph nodes, or by a slow-release combination close to the tumor site. The immune stimulation is thereby focusing on the tumor area, minimizing systemic exposure and thus reducing systemic side effects. Tumor-directed immunotherapy by design can be achieved using bispecific cross-linking-dependent agonistic TNFR antibodies where a tumor-binding part mediates the cross-linking, replacing the need for FcγR-mediated cross-linking. In the absence of tumor cells, these types of bispecific antibodies will not be active, minimizing systemic immune activation and reducing systemic side effects

Tumor-directed immunotherapy is an approach to focus the immune activation to the most relevant part of the immune system (Fig. 1). This concept has also been termed in situ vaccination [2, 3]. The aim of tumor-directed immunotherapy is to activate immune cells that have already homed to the tumor/local lymph node where tumor antigens are present, while minimizing irrelevant activation of the rest of the immune system. Preclinical data suggest that this can reduce immune-related adverse events (irAE). A critical aspect of tumor-directed immunotherapy is that it must be able to generate a systemic anti-tumor response that eradicates distant metastases and induces long-term tumor immunity.

Tumor-directed immunotherapy would allow the use of highly potent immune modulating therapies and combinations without increasing the risk for the patients. In addition to decreasing the risk for inducing toxicity, tumor-directed immunotherapy may reduce secondary systemic anti-inflammatory feedback responses that dampen the anti-tumor immune response. In the case of monoclonal antibodies, tumor-directed immune activation can be achieved by local injection into the tumor area or by targeting the tumor using bispecific antibodies.

In this review, we focus on therapies targeting checkpoint inhibitors and co-stimulatory receptors that facilitate tumor-specific T cell responses through localized immune activation. Cancer vaccines, oncolytic viruses, local injections of cytokines, and Toll-like receptor (TLR) agonists are covered elsewhere [3, 4].

## Cancer immunotherapy results in activation or reactivation of tumor-specific T cells

The ultimate goal of cancer immunotherapy is to generate a strong tumor-specific T cell response enabling effector T cells to find and kill tumor cells, irrespective of localization or number of tumor lesions. Immune checkpoint therapy based on either anti-CTLA-4 or anti-PD-1/PD-L1 blocking antibodies inactivates the brakes on T cells, allowing broad activation of T cells, including tumor-specific T cells. In addition, experimental model systems have revealed that antibodies targeting CTLA-4 deplete Treg in the tumor microenvironment [5]. Although there are currently no clinical data confirming this, ex vivo studies support this proposed mode of action of ipilimumab [6].

CTLA-4 or PD-1/PD-L1 blockade is associated with increased survival in melanoma, renal cell cancer, non-small cell lung cancer, bladder cancer, and Hodgkins lymphoma [7]. In fact, the combination of anti-CTLA-4 and anti-PD-1 therapy appears to be even more effective, albeit at the cost of a higher frequency of irAE. Nevertheless, this has sparked considerable optimism in the cancer immunotherapy field. Antibodies blocking additional checkpoint inhibitors such as LAG-3, TIM-3, and VISTA are currently in early clinical trials.

Strategies targeting checkpoint inhibitors have proven particularly successful in T-cell-infiltrated immunogenic tumors. However, turning non-immunogenic tumors into immunogenic tumors remains a challenge. Co-stimulatory agonistic antibodies may prove to be valuable to this end. Currently, agonist antibodies targeting the co-stimulatory receptors CD40, OX40, ICOS, CD27, GITR, and CD137 are evaluated in the clinic [8–10]. Most of these co-stimulatory receptors are expressed on T cells. In contrast, CD40 is mainly expressed on antigen-presenting cells, such as dendritic cells (DC). Activation of CD40 on DC improves their cross-presentation of tumor antigens and release of IL-12, thereby boosting the number of activated tumor-directed T effector cells.

## Tumor-directed immunotherapy can be achieved by local administration of immune modulating drugs

Use of T cell co-stimulators is often associated with cytokine release. When these agents are combined with checkpoint inhibitors, there is a risk for aggregated toxicity. Tumor-directed immunotherapy may be a way to allow for such combinations while avoiding an increase in frequency and severity of irAE. There are two conceptually different ways to generate a tumor-specific T cell response through localized co-stimulation, i.e., either by local administration of the co-stimulator in the tumor area, or by developing co-stimulators designed to exert their effect predominantly in the tumor microenvironment (Fig. 1).

In the clinical setting, local administration of antibodies targeting checkpoint inhibitors and co-stimulatory receptors in the tumor area has been proposed primarily for the treatment of unresectable tumors or as pre- or postsurgical adjuvant therapy to prevent local recurrence. However, a growing number of studies have suggested that intratumoral injections of antibodies targeting checkpoint inhibitors and co-stimulatory receptors can generate a systemic anti-tumor response and immunity, eradicating also metastases distant to the injection site. Compared to intravenous administration, the intratumoral route may reduce acute as well as overall systemic exposure, and hence reduce the risk of acute reactions such as cytokine release syndrome and late-onset irAE.

### Preclinical studies using tumor-directed immunotherapy

Several studies in animal models have shown that local co-stimulation using agonistic antibodies can drive systemic anti-tumor effects and induce T-cell-dependent anti-tumor immunological memory [11–15]. The most widely studied co-stimulatory target using local drug administration is CD40 where the concept has been demonstrated in multiple models. Such models include virally transduced tumors [12, 13], multiple myeloma, lung cancer [12] as well as bladder tumors [11, 15]. Injection in or near the tumor is critical for generating the systemic tumor effect. This was demonstrated using experimental tumor models with multiple tumors, where the anti-tumor effect was severely impaired when CD40 activating therapy was administered at a site distant from the tumor [11, 13].

One of the critical issues when using local administration is the distribution of the antibody following injection. It has been demonstrated that injection in or near the tumor results in increased accumulation in the tumor-draining lymph nodes and that the systemic Cmax is reduced compared to systemic administration [11]. It has also been shown that increased affinity of the CD40 agonistic antibody results in increased accumulation of the antibody in the tumor area [15].

Moreover, a replication-deficient adenoviral vector expressing CD40 ligand (AdCD40L) has been studied in murine bladder cancer models. Weekly intra-/peritumoral injections of AdCD40L cured subcutaneous and orthotopic bladder tumors as well as distant tumors [16]. This was associated with increased T cell infiltration and generation of cytotoxic T cells [17].

Locally administered CTLA-4 blocking antibodies have also been assessed in several experimental tumor models, including transduced epithelial tumors [18], colon cancer [19], pancreatic cancer [20], and bladder cancer (unpublished data). CTLA-4 is of particular interest in this regard because of the high incidence of irAE associated with systemic CTLA-4 blockade [21].

Agonistic OX40 antibodies in combination with CTLA-4 antibodies and CpG have shown promising activity in experimental lymphoma and breast tumors upon local administration [22]. Further, Palazón et al. [23] showed that local stimulation of 4-1BB using agonistic antibodies may be a promising approach to treat colon tumors and avoid systemic side effects.

### Clinical studies using tumor-directed immunotherapy

Preliminary data from studies of intratumoral ipilimumab in combination with IL-2 (NCT01672450) indicate good tolerability. Intratumoral administration of ipilimumab in combination with TLR9 agonists is also under investigation (NCT02254772), and local administration of TLR agonists has been studied in several clinical trials [24, 25], demonstrating both tolerability and clinical response. Further, a phase I/II study investigating intratumoral administration of tremelimumab in combination with TLR agonist (poly I:C) and systemic PD-L1 blockade has recently been initiated (NCT02643303). In addition, a first-in-man trial of intratumoral CD40 agonistic monoclonal antibody ADC-1013 was initiated in early 2015 (NCT02379741).

Several clinical studies have also been performed using adenoviral vectors expressing CD40 ligand (AdCD40L). A first-in-man phase I/IIa study was conducted with local instillation of AdCD40L in high-grade bladder cancer patients scheduled for cystectomy [26]. Patients received 3 weekly local instillations of the vector, followed by cystectomy. The treatments were well tolerated. Treated bladder tissue expressed CD40L as well as IFNγ and resulted in T cell infiltration and total or partial disappearance of malignant cells in 5/8 treated patients, thereby providing proof of concept [26]. This trial was followed by studies in dogs with spontaneous high-grade malignant melanoma [27]. In total, 19 dogs were treated, resulting in 5 complete and 8 partial responses, 4 stable disease and 2 progressive disease, including effects on distant metastases [28]. More recently, a phase I/IIa study of intratumor AdCD40L in patients with therapy-resistant metastatic malignant melanoma was initiated. Side effects were mild, and local and distant anti-tumor effects were observed in MR-PET imaging. Addition of low-dose i.v. cyclophosphamide resulted in prolonged survival compared to AdCD40L alone [29]. Further, replication-competent adenoviruses can be engineered to exhibit oncolytic, i.e., tumor cell selective killing and immunostimulatory, properties [30].

### Technical aspects of intratumoral administration

Several challenges need to be addressed to bring this concept to the patients. Methods to prevent systemic leakage of the injected antibody using micro- or nanoparticles or emulsions have been described [31, 32]. Further, it is likely that immune cells both in the tumor and in tumor-draining lymph nodes are important, which may affect the requirement of appropriate exposure. The relative importance of the immune cell locations may vary depending on target and tumor type.

Intratumoral administration may be associated with adverse events such as local inflammation, pain, and bleeding. Local inflammation and associated pain could also be a sign of successful immune activation in the tumor area. The risk of bleeding after intratumoral injection is small (probably <1 % of injected tumors). Monitoring of patients for a few hours post-injection into deep tumors is however recommended. Another potential risk is that the immune activator may be mistakenly injected in a blood vessel during intratumoral administration, thus leading to rapid and complete uptake in the circulation with risk of acute cytokine release as a result. This risk is however considered small and can be further reduced by avoiding injections in well-vascularized tumors and by using an intratumoral injection guidance including aspiration prior to injection.

From a practical point a view, it is also important to consider factors such as injection volume, injection speed, diameter of needles, and size of tumors. When administrating intratumorally, the injection volume should be kept low (preferably below 500 µL) to minimize systemic leakage. For tumors larger than 3 cm in diameter, the dose may be administered close to the tumor margin in order to maximize the chance of exposing relevant immune cells. The size of the injected tumors may also affect the response. One hypothesis is that injection into larger tumors, e.g., larger than 5 cm in diameter, may lead to reduced efficacy due to large regions of central necrosis, while injection into tumors smaller than 1 cm may lead to increased leakage of the immune activator from the tumor. Further, the speed of injection may depend upon the density of the tumor tissue and the ease of distributing the study drug solution through the tumor while still allowing the retention of the study drug within the tumor.

Selection of the tumor(s) to inject is primarily driven by feasibility. It could be argued that the primary tumor would offer the best opportunities to direct the response to “trunk” mutations, i.e., mutations that arise early in the tumorigenesis and are shared by the majority of the tumors in the patient, and may be associated with a better response than branch mutations that arise at later stages [33]. However, the quality and quantity of the immune infiltrate in different tumors in the same patient may also affect the choice of tumor to inject.

## Tumor-directed immunotherapy can be achieved by designing drugs to act locally

Another approach to perform tumor-directed immunotherapy is to use immune-activating bispecific antibodies that incorporate tumor-targeting entities or monospecific antibodies that are preferentially active in the tumor milieu. Bispecific antibodies with a tumor-specific binding entity can be administered systemically, localize to the tumor area and thereby mediate tumor-directed immunotherapy.

### Immunocytokines

Immunocytokines consist of a cytokine moiety fused to a monoclonal antibody or antibody fragment [34–39]. The antibody fragment binds to tumor-associated proteins, tumor vascular targets, or targets in the tumor stroma and can redirect the cytokine to the tumor area. The main purpose is to limit the systemic toxicity that is associated with cytokine treatments. Several studies have demonstrated that cytokines indeed can be redirected to the tumor area using this approach. Preclinical studies with immunocytokines using several different cytokines, e.g., TNFα, GM-CSF, IL-2, IL-12, IL-7, IL-15, IL-17, IL-18, IL-10, IFNγ, and IFNα, have generated impressive results with improved anti-tumor effects [34, 35]. However, most of the immunocytokines can be found outside the tumor area, and the increased half-life conferred by fusing an antibody to the cytokine can result in prolonged systemic exposure, which limits the therapeutic window. Intratumoral administration of immunocytokines may be an approach to address this [34, 35]. There are currently a number of ongoing clinical trials using immunocytokines using IL-2, IL-12, and TNF as the cytokine moiety [35]. However, the clinical progress has so far been modest.

### Engineered monoclonal antibodies

Monoclonal antibodies can also be designed to preferentially accumulate in the tumor tissue by increasing the isoelectric point. The pH in tumors is significantly more acidic (6.6–7.0) than that of normal tissues (7.2–7.4). This acidity is primarily due to anaerobic glycolysis in tumor regions subjected to short-term or long-term hypoxia as a result of poorly organized neovasculature with diminished chaotic blood flow, and aerobic glycolysis (the Warburg effect) [36, 38]. Antibodies with higher isoelectric point may be retained in the acidic environment. Another promising approach would be to generate antibodies that preferentially bind their target at lower pH, designing the antibody binding site to depend on the protonated form of histidine residues [37, 39]. Further, a class of proteolytically triggered antibodies engineered to remain inert until enzymatically activated in the tumor microenvironment has been described [40].

### Redirection of T cells using CD3-targeting bispecific antibodies

CD3-targeting bispecific antibodies can be utilized to redirect T cells toward tumor cells. This approach has proven successful, and blinatumomab, a bispecific T-cell-engaging antibody that binds CD19 on B cells and CD3 on T cells, was approved in 2014 for the treatment of relapsed/refractory B cell precursor acute lymphoblastic leukemia [41]. Several T cell redirecting therapies are currently in clinical development against solid tumors [42]. While CD3-targeting bispecific antibodies effectively kill tumor cells, they depend on polyclonal T cells for tumor killing, and they do not directly promote activation of T cells that specifically recognize tumor-associated antigens. This approach may therefore be less effective in inducing a long-term tumor immunity compared to immunotherapies targeting checkpoint molecules.

### Bispecific antibodies selectively activated in the tumor microenvironment targeting TNFR-SF members

Another approach to achieve tumor-directed immune activation is to utilize bispecific antibodies that are activated upon binding to the tumor cell. To this end, cross-linking-dependent agonistic antibodies targeting the TNFR-super family may prove advantageous. Agonistic IgG1 antibodies targeting, e.g., CD137, CD40, or OX40, have limited agonistic effect without cross-linking provided by FcγR on adjacent cells [43–45]. Cross-linking may however be mediated by entities other than FcγR, such as a tumor-associated antigen. It is thus possible to generate bispecific anti-TNFR superfamily antibodies, where FcγR binding has been removed and replaced by tumor-associated antigen binding. Such compounds would only be active when cross-linked by the tumor-associated antigen in the tumor area. Stimulation of TNFR superfamily members is known to induce tumor-specific memory T cell responses [46, 47], and accordingly, this class of bispecific antibodies may have the potential to induce long-lasting tumor immunity.

Tumor-directed immunotherapy based on the above concept was first demonstrated using the natural ligand to CD137 (CD137L) fused to a tumor-targeting scFv [48, 49]. This concept may however be extended to any antibody activated after cross-linking, such as agonistic antibodies targeting TNFR superfamily members.

## Concluding remarks

Tumor-directed immunotherapy, or in situ vaccination, is currently being evaluated in both preclinical studies and clinical trials. This approach has the potential to improve the response rate and increase the number of tumor indications that respond to immunotherapy, while limiting adverse side effects. Efforts are ongoing to generate next-generation immunotherapeutic drugs specifically designed for tumor-directed immunotherapy, and we predict that this field will grow substantially during the coming decades.

## Abbreviations

AdCD40L: Adenoviral vector expressing CD40 ligand
Cmax: Peak plasma concentration of a drug after administration
CTLA-4: Cytotoxic T-lymphocyte-associated protein 4
FcɣR: Fc gamma receptor
ICOS: Inducible T cell co-stimulator
irAE: Immune-related adverse events
LAG-3: Lymphocyte-activation gene 3
PD-1: Programmed cell death protein 1
PD-L1: Programmed death-ligand 1
TIM-3: T cell immunoglobulin and mucin-domain containing-3
Treg: Regulatory T cells
VISTA: V-domain immunoglobulin (Ig)-containing suppressor of T cell activation
